# Concept Libraries for Repeatable and Reusable Research: Qualitative Study Exploring the Needs of Users

**DOI:** 10.2196/31021

**Published:** 2022-03-15

**Authors:** Zahra Almowil, Shang-Ming Zhou, Sinead Brophy, Jodie Croxall

**Affiliations:** 1 Data Science Building Medical School, Swansea University Swansea, Wales United Kingdom; 2 Centre For Health Technology Faculty of Health University of Plymouth Plymouth United Kingdom

**Keywords:** electronic health records, record linkage, reproducible research, clinical codes, concept libraries

## Abstract

**Background:**

Big data research in the field of health sciences is hindered by a lack of agreement on how to identify and define different conditions and their medications. This means that researchers and health professionals often have different phenotype definitions for the same condition. This lack of agreement makes it difficult to compare different study findings and hinders the ability to conduct repeatable and reusable research.

**Objective:**

This study aims to examine the requirements of various users, such as researchers, clinicians, machine learning experts, and managers, in the development of a data portal for phenotypes (a concept library).

**Methods:**

This was a qualitative study using interviews and focus group discussion. One-to-one interviews were conducted with researchers, clinicians, machine learning experts, and senior research managers in health data science (N=6) to explore their specific needs in the development of a concept library. In addition, a focus group discussion with researchers (N=14) working with the Secured Anonymized Information Linkage databank, a national eHealth data linkage infrastructure, was held to perform a SWOT (strengths, weaknesses, opportunities, and threats) analysis for the phenotyping system and the proposed concept library. The interviews and focus group discussion were transcribed verbatim, and 2 thematic analyses were performed.

**Results:**

Most of the participants thought that the prototype concept library would be a very helpful resource for conducting repeatable research, but they specified that many requirements are needed before its development. Although all the participants stated that they were aware of some existing concept libraries, most of them expressed negative perceptions about them. The participants mentioned several facilitators that would stimulate them to share their work and reuse the work of others, and they pointed out several barriers that could inhibit them from sharing their work and reusing the work of others. The participants suggested some developments that they would like to see to improve reproducible research output using routine data.

**Conclusions:**

The study indicated that most interviewees valued a concept library for phenotypes. However, only half of the participants felt that they would contribute by providing definitions for the concept library, and they reported many barriers regarding sharing their work on a publicly accessible platform. Analysis of interviews and the focus group discussion revealed that different stakeholders have different requirements, facilitators, barriers, and concerns about a prototype concept library.

## Introduction

### Background

Health care systems are becoming more digitally focused rather than paper-based and are moving to the use of electronic health records (EHRs) [1]. This means there is a large amount of electronic patient data that can be moved and linked together into safe data repositories to enable researchers and data analysts to query and examine these data effectively [2-5]. The growing availability of electronic patient data offers health care practitioners increased opportunities for secondary use of EHR data to improve the quality of care and research [6-8]. However, the present literature does not describe the barriers that make the use of data and deidentification processes difficult nor does it focus on users’ practical needs for data linking [9]. A study observed that “One of the fundamental steps in utilizing this EHRs data is identifying patients with certain characteristics of interest (either exposures or outcomes) via a process known as electronic phenotyping” [10]. Phenotyping is the process of extracting phenotypes from clinical data using computer-executable algorithms [11], and phenotypes are “the measurable biological, behavioural and clinical markers of a condition or disease” [12]. Phenotypes might be as simple as patients with type 2 diabetes or as complex as patients with stage II prostate cancer with urinary urgency but no indications of urinary tract infection [10].

There has been an annual rise at a rate of approximately 20% in primary care research using EHRs in the United Kingdom, which gathers data on general practice from the following databases [13]: Clinical Practice Research Data Link [14], The Health Improvement Network [15], QResearch [16], and Secured Anonymized Information Linkage (SAIL) [17]. However, with different data sets (eg, hospital, general practice, or emergency care), defining a condition is still very subjective, as there are many phenotyping algorithms for identifying the same condition (eg, there are currently 66 ways of defining asthma using routine health data) [18], and interpretation or manipulation of data often requires knowledge of complex programing languages, such as SQL [4]. This means that EHRs are still not accessible to many as their use requires specialized programing skills.

One of the most important factors for reproducible research is the availability of clinical codes in EHR-based research because researchers, clinicians, and health informatics professionals often use them to identify the target population and their specific conditions, known as phenotyping [8,19]. If researchers do not publish the code lists they used (eg, how they were established and the accurate phenotype definitions along with the original research using them), then an essential component of these studies is missing. In the absence of clinical code lists, data analysts would be unable to identify patients with or without conditions [19], and researchers would not be able to compare studies effectively. Even though code lists are available in some studies, researchers often encounter difficulties in retrieving relevant data from code lists created for another research project. Moreover, in specific uncommon conditions, minor errors in the selection of code lists may lead to misclassification of large numbers of patients, leading to biased results [20]. Although using previously developed phenotyping algorithms is often of interest to researchers in many studies, there are many challenges associated with reusing and replicating them effectively [21]. Therefore, it is extremely difficult to assess the validity and transparency of EHR-driven studies [22].

Although researchers request better transparency in sharing clinical code lists [23,24], they face difficulties in obtaining comprehensive code lists from EHR-based research. Although, there are currently no obligations from journals and funding parties to publish code lists, the Strengthening the Reporting of Observational Studies in Epidemiology and Reporting of Studies Conducted Using Observational Routinely Collected Health Data initiatives encourage transparency and open access to publicly available EHR-based research [25-27]. To address these challenges, different data linkage centers in the United Kingdom and other countries, such as Canada, have developed data portals for phenotypes (concept libraries), such as ClinicalCodes.org [22], Clinical Disease Research Using Linked Bespoke Studies and Electronic Health Records (CALIBER) data portal [4], and the Concept Dictionary at the Manitoba Centre for Health Policy [28]. Building web-based concept libraries enables data analysts, researchers, and clinicians to upload and download lists of clinical codes, update previous code lists, and share clinical code data across platforms, which would improve the validation of EHR-based research [22].

### Objectives

This study aims to explore the needs of various users, including researchers, clinicians, machine learning experts, and managers, to develop a data portal for phenotypes (a concept library) and to examine why existing concept libraries are not widely used.

## Methods

### Design

A qualitative study using one-to-one interviews and a focus group discussion was conducted. We recruited a small purposive sample for in-depth one-to-one interviews in the first phase because it allows us to obtain substantial information from a small number of participants while also providing insight into their different viewpoints, needs, and experiences with concept libraries. In the second phase, we recruited a larger sample of participants for the focus group discussion to improve the generalizability of the results. The inclusion criteria were to recruit potential users of concept libraries from various disciplines, including researchers, clinicians, machine learning experts, and managers who conducted studies using routine data generated by data linkage repositories.

For this study, we adopted a semistructured approach. We created semistructured interview questions based on the Krueger and Casey format [29], which included introductory, flow, key, and final questions to be used in one-to-one interviews (Table 1). We also created a list of 10 questions based on the objectives of this study for the focus group session. The purpose of the questions was to generate thoughtful and thorough responses from the participants; therefore, closed-ended questions (eg, yes or no) were avoided. The interviews and the focus group discussion were audio recorded and transcribed verbatim, and 2 thematic analyses were performed using the 6 steps of Braun and Clarke to identify the themes and subthemes [30].

**Table 1:** One-to-one interviews’ questions guide.

Introductory questions	Follow questions	Key questions	Final questions
To improve repeatable research in Swansea, a team of developers is developing a prototype concept library. This is a portal that allows access to the read codes or International Classification of Diseases–10 codes to identify conditions. Do you think this will be a helpful resource? Is the concept library a good idea that we should continue to develop?	Do you know about other already existing concept libraries? What do you think about them? Something like this exists at UCL^a^ called CALIBER^b^. Have you seen CALIBER? Have you used it?	Do you prefer to use ready-made algorithms or to have access to them to modify them? In your opinion, how should codes and algorithms be validated, and should they be validated? (Why should or should not?) There are often different versions of a diagnosis (eg, highly specific and suspected or likely cases). Do you think we need to collect and validate the best two versions of a diagnosis (specific or suspected)? Or do you think we should put all possible methods of identifying a condition, valid or not, and allow the researcher to choose?	What are your requirements for the concept library for it to be helpful and user-friendly? What developments would you like to see to improve repeatable research using routine data?

^a^UCL: University College London.

^b^CALIBER: Clinical Disease Research Using Linked Bespoke Studies and Electronic Health Records.

### Data Collection

The first author asked 6 participants from a variety of disciplines, including researchers (3/6, 50%), a clinician (1/6, 17%), a machine learning expert (1/6, 17%), and a senior research manager (1/6, 17%), at Swansea University and Cardiff University to participate in one-to-one interviews by email. The invitation email specified the aim and purpose of this study, the duration of each interview (30 minutes), and the location of the interviews, which might be their offices or a convenient and private location on the Swansea University campus.

Semistructured interview questions, which follow the structure proposed by Krueger and Casey [29], were used (Table 1). The structure of the interview questions consisted of introductory, flow, key, and final questions. The purpose of the introductory questions was to help the participants talk freely about their overall experiences. The flow questions were designed to create a smooth transition to the key areas that the authors intended to explore. The final questions were designed to summarize the interview and ensure that the participants did not have further comments [31].

Before conducting the interviews, the first author explained the purpose of the research and what it involved, and at the beginning of each interview, participants received additional verbal and written information about the research project. The interviews were conducted at Swansea University Medical School in a place selected by the participants (eg, their office). After 5 interviews, no new themes were observed and interview 6 confirmed that no new themes emerged. The interviews were audio recorded and transcribed verbatim. Thematic analyses were then performed using the 6 steps of Braun and Clarke to identify the themes and subthemes [30].

All researchers working with the SAIL databank, a national eHealth data linkage infrastructure in Wale (N=34) were invited by email to participate in the focus group discussion, and 14 (14/34, 41%) researchers attended the focus group discussion. In total, 2 focus group discussions, each of which had 7 (7/14, 50%) participants, were held for 2 hours by 2 moderators (ZA and SB), who used the same set of semistructured questions to perform a SWOT (strengths, weaknesses, opportunities, and threats) analysis for the current system for phenotyping and the proposed concept library. We used a SWOT analysis tool in this study because it enabled the participants to discuss what they liked (strengths), what advantages would be gained (opportunities), and what problems (weaknesses) and issues (threats) they felt needed to be tackled. Although the 2 moderators used the same set of questions, the order of the questions was adjusted to the needs of each group.

At the beginning of the focus group discussion, the first author gave a brief presentation about concept libraries, including defining concept libraries, explaining their potential uses, and mentioning examples of some of the existing concept libraries in the United Kingdom. A second presentation about the Swansea University prototype concept library was then given by one of its developers. Feedback from the participants was sought concerning their perceptions of the concept library’s needs and their evaluation of the strengths and limitations of the proposed concept library. Participants’ perceptions of existing concept libraries, as well as their assessment of the proposed concept library’s strengths and limitations, were explored using the following set of semistructured questions:

- What are your thoughts regarding the proposed data portal for phenotypes (a concept library) when it rolls out?
- Do you think this is worth doing? Would you value this?
- Has anybody used existing concept libraries? What have you experienced with them?

Let us talk now about your current system for phenotyping:

- What do you do? What are your methods?
- Are you happy with them? Or what would you like differently?
- What are your thoughts on this plan (building a concept library)?
- Would you use it? Would you share your phenotypes and your phenotyping algorithms?

If you do not want to share your work:

- Can you tell us why? And what motivates you to share it with others?
- Of all the things we have discussed, what is most important to you?
- Is there anything we should have talked about but did not?

The goal of using the SWOT analysis was to identify positive factors that operate together and the potential difficulties that must be identified and solved. During the focus group discussions, participants expressed their own opinions and listened to the opinions of others. As the discussions progressed, participants began to ask questions of one another and share similar experiences. This increased the depth of the conversation. The SWOT analysis gave us a full picture of views and experiences of concept libraries by the participants, making this a holistic evaluation with the ability for participants to hear and comment on each other’s responses. Textbox 1 presents a summary of the SWOT analysis in the current system for phenotyping and the proposed concept library. The 2 focus group discussions were audio recorded and transcribed verbatim. Thematic analyses were then conducted using the 6 steps of Braun and Clarke to discover the main themes and subthemes (Table 2).

**A summary of a SWOT (strengths, weaknesses, opportunities, and threats) analysis of the current system for phenotyping and the prototype concept library.**

**SWOT analysis**

**Strengths**

- Concept libraries provide researchers with a good starting point.
- Publicly available code lists may provide researchers with a history of a particular area of research, such as asthma.
- Referencing previously published lists of codes enables researchers to demonstrate a rationale for using such lists of codes.
- Using research methods developed by others that match the researchers’ interests could result in significant time saving.
- Collaboration among researchers is facilitated through sharing and using research methods such as code lists.

**Weaknesses**

- Searching for and reusing phenotypes and codes is a time-consuming and labor-intensive process.
- There are various lists of codes for each phenotype definition.
- The list of codes chosen by clinicians varies significantly.
- A large number of previously developed code lists could not be repeated.
- Reusing other researchers’ data requires programing knowledge such as SQL.
- Some of the ready-made phenotyping algorithms may not be very useful in terms of their general purpose.
- Some existing concept libraries have limited user interfaces.
- Some existing concept libraries are not user-friendly.
- It is unclear who is accountable for the quality of the uploaded codes in concept libraries.
- The validity of the content of concept libraries is unclear.

**Opportunities**

- Concept libraries must provide user documentation.
- Concept libraries must provide users with training.
- Transparency in sharing the whole approach used to create the code lists is required.
- Establishing a standardized way of defining each specific condition to facilitate comparisons of research outcomes across the United Kingdom.
- Creating a specialized library that stores code lists of a specific condition within a specific set of patients, such as a concept library specializing in chronic conditions in children.
- Creating a concept library that engages a wide variety of users (ie, is easily understandable by clinicians but has some advanced features such as programing skills for more expert users).

**Threats**

- The inconsistency of data across various databases makes data reuse difficult.
- Lack of confidence in the quality of the list of codes developed by other researchers if they are not cited.
- Access to code lists is limited as some researchers do not publish them alongside their studies.
- Different research outcomes result from a lack of access to a list of codes created by other researchers.
- Data sharing may be inhibited if there are no returns, such as referencing and acknowledgment.
- Concerns about ownership rights discourage data sharing (eg, methods could be used as their own by other researchers before publication).

**Table 2:** Presentation of the themes and subthemes of the one-to-one interviews.

Themes				Examples of participant narratives
**Theme (1): previous opinion of a prototype concept library**				
	Positive			“If there’s a way of doing that already that is set up and is validated and is consistently applied that would be an amazingly useful resource” (researcher 2).
	Neutral			“It will be helpful, but it needs to be extended. If they want to build something like this, and it is effectively working as a library, you need two things to be happened: (1) people are happy to feed in their constructs so it builds up, and (2) a useful library, easy to go, to browse, and to borrow phenotypes definitions” (a clinician).
	Negative			None
**Theme (2): requirements of a prototype concept library**				
	**Usability**			
		Simplicity	“Simple plain English not in SQL or python” (a clinician).	
		Searching ability	“What is the type of search engine? Is it a search engine that just does disease phenotypes or also does the health status phenotypes or risk factor phenotypes, symptoms phenotypes?” (a clinician).	
		Data quality	“It’s really just about transparency and documentation. So, anybody can effectively do anything that can be turned into a reproducible research output. The barriers are usually not enough time to comment and document it properly and then not enough quality assurance” (a senior research manager).	
		Sharing ability	“It would be very useful to share the knowledge about codes such as read codes, ICD 10 codes, or OPCS codes, and share ideas and concepts between other users that will save lots of time” (researcher 3).	
	**Sustainability**			
		Interoperability	“How interoperable it is with other systems because the major failure of most of these systems is that they’re not interoperable, so people don’t use them” (a senior research manager).	
		Accessibility	“So, from a group like myself, or me as a user, we would probably like direct access to the underlying data it stores. So, whether that’s through something like SQL directly, or something like that through a statistical package, because where we do lots of bulk type work” (a senior research manager).	
		Analyzability	“I wanted to look at all health codes of my study population. Then, through machine learning, like feature selection, I tried to identify the most important list of codes, which are associated with the popular health conditions” (researcher 1)*.*	
**Theme (3): user experience of existing concept libraries**				
	Aware (used them)			“Yes, so with QOF, we definitely used QOF codes a lot, because obviously going back to the quality assurance question, they’d been assured so that the NHS can use them for remuneration of money and payments. With other systems, we tend to look online to see CALIBER of things with us, then yes we have used outputs from those systems before” (a senior research manager).
	Aware (not used them)			“No. I have not used any of these things before so I think there is CALIBER and I think, is that part of what was set up within the previous Farr institute? so I am aware that some of these exist but I haven’t looked into them before” (researcher 2)*.*
	Not aware			None
Theme (4): user’s recommendation to improve repeatable research				“If we want reproducible research, we have to all be using these resources in a similar way or at least we need to be able to understand what previous projects have done. It is about setting things out clearly. Clear definitions, clear sets of codes that people can then either use themselves or build on I think” (researcher 2)*.*

### Data Analysis

The interviews and the focus group discussion were analyzed separately following the analysis approach by Braun and Clarke [30]. The transcripts of the interviews and the focus group discussion were read several times, and then the initial codes were grouped into themes and subthemes using a qualitative data analysis software (NVivo, QSR International) [30,32]. ZA had read all the transcripts, and SB read a sample of the transcripts. They independently identified the themes and subthemes, then met regularly to compare them and reach an agreement on what was being done. Themes and subthemes were discussed with respect to their relevance to the research question in the data collected. They critically reviewed the themes again to determine their primary meanings, and similar initial themes were combined into one theme. They discussed the definitions of the relevant themes in the research questions and applied appropriate names to describe each in this study. Textbox 2 provides further description of the thematic analytic steps.

**The 6 thematic analytic steps used for this research.**

**Thematic analytic steps**

**Self-familiarizing with the data**

- ZA transcribed half of the audio recordings from the interviews (3/6, 50%). The other half of the audio recordings from the interviews (3/6, 50%) and the audio recordings from the focus group discussion were transcribed by professional transcribers. During this phase, ZA read all the interview and focus group discussion transcripts several times, and SB read samples of them. ZA and SB considered all the topics discussed by the participants, recorded notes on these topics in the transcripts, and then organized them in a note book.

**Creating initial codes**

- After familiarizing themselves with the data, ZA and SB worked independently to identify initial codes from the transcripts that summarized what was said during the interviews and focus group discussion. They organized the identified codes into meaningful groups using qualitative data analysis software (NVivo, QSR International). They used the same coding procedure for all the transcripts.

**Searching for themes**

- ZA and SB started interpreting the initial codes using their extracted data, and they began grouping the codes with similar meanings together. Using the NVivo software (QSR International), the initial codes were then sorted and labeled into themes and subthemes depending on the meaning or relations shared by the codes.

**Revising themes**

- ZA and SB critically reviewed and refined themes against the data several times to determine their core meanings, and similar initial themes were combined into one theme. To reach an agreement, themes and subthemes were discussed in terms of their relevance to the research question.

**Defining themes**

- Each of the themes identified in the previous steps was named and defined by ZA and SB. They used the initial labels created for the themes to provide appropriate names that describe the meaning of the themes in this study. ZA and SB defined each theme based on the content and meaning of their codes, and they examined these definitions in relation to their relevance to the research questions.

**Writing up the report**

- After defining and naming the themes, ZA and SB began writing the findings for this manuscript. They used quotes from the participants’ responses that related to the themes and the research question to illustrate the findings.

### Ethics Statement

Ethical approval to conduct the research was approved by the Research Ethics Sub-Committee of Swansea University, project reference number 2019-0007.

## Results

### Interviews With Users

#### Overview

In total, 6 one-to-one interviews were conducted, and each interview lasted for approximately half an hour. The analysis of the interviews resulted in 4 main themes, with several subthemes (Table 2). The four main themes are as follows:

1. Previous opinion of a prototype concept library
2. Requirements of a prototype concept library
3. Experience of existing concept libraries
4. Recommendations to improve repeatable research

#### Previous Opinion of a Prototype Concept Library

The majority of the participants were positive about the prototype concept library and felt that a concept library in principle was a very helpful resource for conducting repeatable research. A machine learning expert mentioned that a concept library will be an extremely useful resource because read codes from general practice and International Classification of Diseases (ICD)–10 codes from hospitals are the most common data items that machine learning experts would like to use most often. They use data linkage repositories to extract the necessary data for machine learning in public health studies, and they use the codes to extract the data from the repositories. Researcher 3 said, *“*It would be very useful to share the knowledge about codes such as read codes, ICD 10 codes, or OPCS codes, and share ideas and concepts between other users that will save lots of time. It is useful to use verified codes,*”* and researcher 2 stated, “If there’s a way of doing that. Already that is set up, and is validated and is consistently applied, that would be an amazingly useful resource.”

However, 2 participants (a clinician and a senior research manager in health data science) were not sure about the effectiveness of the prototype concept library because they felt that users had to engage with it for it to be useful and they were not sure how well users would engage: *“*There is potential that it could be useful as a tool. It will kind of come down to how usable it is, how flexible it is, how well it's maintained, how much of the community uses it” (a senior research manager).

#### Requirements of the Prototype Concept Library

The participants mentioned several requirements they would like to see in the prototype concept library. For example, they stated that the concept library needed to have high usability. This means that it needs to be simple and easy to use by naïve users: “It should be simple enough, within one or two clicks; we can find the required data, but also should contain advanced expert features (R, SQL, or Python programing languages) to extract, include, or exclude codes necessary for their studies” (researcher 3) and *“*Like, in one of my previous projects, I looked at, from a machine learning perspective, I wanted to look at all health codes of my study population. Then, through machine learning, like feature selection, I tried to identify the most important list of codes, which are associated with the popular health conditions” (researcher 1)*.* They also stated that the concept library should have a good search engine so that they can easily find the phenotypes and phenotyping algorithms they want to use. A clinician inquired, “What is the type of search engine you are developing? Is it a search engine that just does disease phenotypes? or also health status phenotypes, risk factor phenotypes, or symptom phenotypes. For example, I am looking for diabetes, but I may also be looking for smoking or alcohol consumption, or symptoms like pain or cough. So, how big is the enterprise and how do you search for what are the appropriate terms? Discussion is needed to know what is it?”

In addition, the participants stated the following requirements:

1. Include the data sources used (eg, codes from general practice, hospital [ICD and Systematized Nomenclature of Medicine], and British National Formulary medication), a general clinical code list for comparison, lists of ontologies along with their variances and versions, and a description of how codes were established: “It is about setting things out clearly. Clear definitions, clear sets of codes that people can then either use themselves or build on, I think” (Researcher 2).
2. Have a clear phenotyping algorithm labeling convention for search engines. A clinician stated,“What do you search on? Thought about what do you call these phenotypes? Is there a consistent in calling them? For example, Type II diabetes, or insulin dependent diabetes” and researcher 1 stated, "So, first of all, for the code reference library, two things are always there in my mind. It’s in my opinion again. Number one, they should be validated. Secondly, they should be correctly labelled.”
3. Specify why a particular phenotyping algorithm was developed (eg, definite disease or probable/suspected condition definitions): “When I have an algorithm, I want a field that tells me the purpose of the algorithm, a brief description of what the algorithm is intended to do” (a clinician).
4. Illustrate the logic model category used to create phenotyping algorithms (ie, code lists, inclusion or exclusion factors, and clinical or machine learning approach used). “Is this just a code list of inclusion factors? And or exclusion factors? Or is it static? Does it have a tampered relationship? So, some algorithms are present or absence of conditions, some required a tampered dependence. In the logic model categories: Is this a clinically derived algorithm from experts’ views or for instance that machine learning derived algorithms” (a clinician).
5. Use ready-made phenotyping algorithms that can be modified to fit the needs of their research. All participants agreed that if they had to create their own phenotyping algorithms because ready-made phenotyping algorithms could not be modified, they needed an easy approach to use a code list in the concept library.

There was an issue regarding how to validate phenotyping algorithms, and most participants expressed their preferences for using all possible methods of identifying a condition, valid or not, to allow the researcher to choose the phenotyping algorithms according to their research requirements: “So, there is no right answer for that because it’s going to be very dependent on your research question, your study group, and your study design. So, once again, if the concept tool is going to match multiple different use cases, it’s going to need to accommodate for those different types of study design” (a senior research manager). Sharing phenotyping algorithms needed to be easy and not time-consuming, and some felt there needed to be some recognition of their work before they would give their codes. Finally, a concept library must be interoperable with other products or systems: “How interoperable it is with other systems, because the major failure of most of these systems is that they’re not interoperable, so people don’t use them” (a senior research manager). Most participants wanted the source code (eg, the SQL code for the phenotyping algorithm itself) to be available in a downloadable machine-readable format to be able to access it using specific programing languages such as R, SQL, or Python.

#### Experience of Existing Concept Libraries

All participants stated that they were aware of some existing concept libraries, such as CALIBER and ClinicalCodes.Org (both in the United Kingdom), but most of them did not use them. The reasons given for not using them were that they already had their own self-made concept libraries (eg, concepts they have used before) or the available concept libraries did not provide phenotyping algorithms that fit their studies. For example, a machine learning expert mentioned the reasons for not using two of the existing concept libraries, namely the Concept Dictionary at the Manitoba Centre for Health Policy in Canada and CALIBER in the United Kingdom were, “Canadian systems provide Canadian data for their studies, CALIBER is specific for cardiovascular disease and does not have many concepts in it.” Conversely, 2 of the participants mentioned that they used some existing concept libraries to extract and develop phenotyping algorithms for their studies: *“*We definitely used QOF codes a lot, with other systems, we tend to look online to see CALIBER, we have used outputs from those systems before” (a senior research manager).

#### Recommendations to Improve Repeatable Research

The participants suggested the following recommendations to improve repeatable research output using routine data:

1. There should be a drive for more transparency in research methods documentation, such as publishing complete phenotype definitions and clear code lists. A senior research manager stated, “It’s really just about transparency and documentation. So, anybody can effectively do anything that can be turned into a reproducible research output,” and researcher 2 said, “If we want reproducible research, we have to all be using these resources in a similar way or at least we need to be able to understand what previous projects have done. It is about setting things out clearly. Clear definitions, clear sets of codes that people can then either use themselves or build on, I think.”
2. Providing opportunities for researchers to collaborate rather than working in isolation, “The barriers are usually not enough time to comment and document it properly and then not enough quality assurance. So, if there was more time and or more availability of those kinds of opportunities for people to collaborate rather than doing things in isolation, there's almost all the research we do here could be turned into a reproducible type of output” (a senior research manager).
3. Develop a concept library that enables researchers to begin classifying population outcomes using uniform codes: “I think that a resource like this is a very good step in the right direction because I think what people need to start doing is using consistent codes in order to identify conditions or outcomes within populations” (researcher 2).
4. Provide validated phenotyping algorithms that researchers can use directly to avoid duplication, with the ability to modify them to meet their own research needs: “For each project, it always has some specific requirement which is unique, which is not common. There are some things which are common, and there are a few things which are very unique. So, we need to have some algorithms which we can just use to, you know, just to avoid the duplication, but also, we need to have control of the algorithms, so that we know only that these bits are going to be different for this project, so I'm going to replace, change, modify this bit, and we'll run it” (researcher 1).

### Focus Group Discussions

#### Overview

Of the 34 invited researchers, 14 (41%) attended the focus group discussion. These participants were researchers (14/34, 41%) from Swansea University who were working with the SAIL data in the Data Science Building. Of the 14 participants, 5 (36%) were female participants and 9 (64%) were male participants. Furthermore, 6 (43%) participants were PhD holders, 6 (43%) were Master’s degree holders, and 2 (14%) were Bachelor’s degree holders (Table 3).

**Table 3:** A summary of general information on the participants in the focus group discussions (N=14).

Parameters	Information
Current job position, n (%)	Data scientist, 13 (93) Financial planner, 1 (7)
Sex, n (%)	Female, 5 (36) Male, 9 (64)
Education, n (%)	PhD degree, 6 (43) Master’s degree, 6 (43) Bachelor’s degree, 2 (14)
Research interests	Data scientists Concept libraries Repeatable research with large health data Phenotyping and code lists of cancer disease Respiratory disease Algorithm or reusable codes development Asthma Collaboration in research methods Data analysis Machine learning Arthritis Health informatics Musculoskeletal disorders Healthy aging Gut—brain axis Neurodegenerative conditions Statistical methods Epidemiology Cancer Financial planners Intervention between primary care and secondary care and how they interact

The focus group discussion was held for 2 hours to perform a SWOT analysis of the current system for phenotyping and the proposed concept library, which was recorded and transcribed, and thematic analysis was conducted on the transcripts, which resulted in the identification of the following seven main themes:

1. Facilitators for and barriers to participants’ contributing their research methods
2. Facilitators for and barriers to participants’ use of other researchers’ methods
3. Participants’ concerns about the prototype concept library
4. The requirements of the participants for the prototype concept library
5. Participants’ recommendations to improve repeatable research
6. Participants’ perceptions of their current phenotyping system
7. Participants’ use and perceptions of existing concept librariess

#### Facilitators and Barriers to Participants’ Contributing Their Research Methods

##### Facilitators

Several facilitators were identified by participants as motivators for them to share their work (eg, phenotyping algorithms and code lists). Many participants stated that being credited appropriately (eg, receiving citations from other researchers) would motivate them to share their work: “If whoever’s using it acknowledges it’s use in whatever they publish, at least you’re getting some recognition*”* (data scientist 8) and “If there were DOIs attached to the code list of algorithms, when people are publishing, there’s an incentive for putting it on there, because they’re able to demonstrate the impact their work has had” (data scientist 4).

Some participants stated that communicating with their research team would encourage them to organize team resources and discuss research findings from other researchers who used their code lists. However, improving research opportunities, increasing academic achievement, and sharing knowledge through collaboration with other researchers working in the same organization would motivate some of the participants to share their work: “I think there’s benefit to the organization, and there has to be benefit to the people contributing to it” (data scientist 4). In general, researchers work in an organization (eg, a university or a research institute), and they work hard to improve the research outcomes of their organization. Some participants stated that advancing the research base and saving other researchers’ time and effort would stimulate them to share their work: “Surely if you’ve done something you think really worthwhile, you want other people to use it, as well, because then that furthers the research” (data scientist 6).

##### Barriers

On the other hand, the participants pointed out several barriers that could inhibit them from sharing their work (eg, phenotyping algorithms and code lists) with other researchers. Some participants argued that it is easy to build a phenotyping algorithm that fits exactly their needs, but it is more challenging to develop a general one, so it can be used by others (eg, many clinical researchers have created phenotyping algorithms for particular research, and these algorithms are difficult to generalize).

Several participants mentioned that a lack of return for their hard work (eg, not receiving any credit from others, such as referencing when they reuse their data) would prevent them from sharing their work: “How do you enforce that people are going to give you credit? It doesn't happen sometimes, when referencing, saying where they got it from. You’ve just got to hope they do” (data scientist 11). Some participants were worried about their intellectual rights (eg, if they shared their methods such as phenotyping algorithms before publication, other researchers would use them as their own)*.*

#### Facilitators and Barriers to Participants’ Use of Other Researchers’ Research Methods

##### Facilitators

The participants mentioned several facilitators that would encourage them to reuse research methods developed by others, such as the following:

1. Using existing code lists can save them a lot of time and effort, which they frequently spend creating new code lists from scratch: “It’s the first stage of every single process, and we tend to get two or three months of work, until we get to that final code list, and we can now start looking at the cases” (data scientist 10).
2. Reusing available data, such as code lists, is a good place to start for researchers (for example, they can use them to examine new ideas and gain new insights):“Having code lists would be such a help, to get you started. They always want things like BMI and weight and height. There are hundreds of codes for those. The smoking codes, having a list, even if you don’t use the algorithm that they’ve developed, is a huge bonus” (data scientist 12).
3. Using the work of others as a reference to compare research outcomes, and researchers want to prove that there is a basis for the use of such codes.

##### Barriers

Conversely, the participants pointed out several barriers that could inhibit them from reusing methods developed by other researchers such as the following:

1. Poor data quality discourages researchers from reusing it: “You could upload complete garbage” (data scientist 1).
2. Some phenotyping algorithms will not work outside the population in which they were developed. For example, code developed in Canada may not be relevant to finding conditions in general practitioner data in the United Kingdom: “Yes, it works in their population, because where they’ve trained it.” (data scientist 5). .
3. Whether the data are useful to researchers plays an important role in the decision to reuse them (eg, researchers would not use a phenotyping algorithm if its general purpose did not match their interests): “Yes, a general-purpose algorithm may or may not be very useful to have it to see what they’ve done, but you may not use it” (data scientist 12).

#### Participants’ Concerns About the Prototype Concept Library

When researchers decide where to deposit, share, and reuse data, they prefer to use approved concept libraries: “Is it going to be approved?” (a financial planner). Moreover, some participants stated that it is not clear who is responsible for the quality of the phenotyping algorithm, if this is the responsibility of the developers running the concept library or the responsibility of the researchers uploading the phenotyping algorithms: “If people send the codes, the onus of the quality of that code list you would still want to be the responsibility of the researcher to be submitting worthwhile codes. You don’t want to then be the guardian of the quality of the code list. You still need to know where the responsibilities lie*”* (data scientist 4)*.* Researchers do not want to upload phenotyping algorithms if they could be *blamed* for flaws, and health informatic developers do not want to take responsibility for the phenotyping algorithms that were uploaded.

The participants expressed their concerns about the completeness rate of the phenotyping algorithms. They would like to know the percentage of the gap to be considered when using a phenotyping algorithm from the prototype concept library: “What is the completeness rate? For certain things, we know there are gaps. If the gap is 20%, is that something I should be including in any algorithm I'm considering?” (data scientist 8). In addition, there has been a question as to whether codes need to be peer reviewed so that quality is evaluated.

#### Requirements of the Participants for the Prototype Concept Library

##### Usability

1. Learnability: Some participants said they would like the concept library to be easily understandable by clinicians, who acknowledge the clinical definition of the code lists with little technical skills to simply point and click the selected code lists, whereas other participants requested the availability of advanced functions to be used by expert users: “The concept library should be easy. Someone needs to train us” (data scientist 9).
2. User documentation: A collection of well-defined task-oriented documentation for users was required by some participants. They want a user documentation that consists of clear, step-by-step instructions on how to use the concept library and gives examples of what the user can see at each step (eg, screenshots would be useful): “Concept library should have some documentation” (data scientist 9).
3. Data quality: Some participants required the availability of a consistent method for identifying each specific condition to ensure that what researchers are doing is compatible within their immediate team but also within the broader research community in the United Kingdom to facilitate a comparison of research outcomes. Other participants stated that they needed a predefined list and a uniform approach describing how to use existing codes of additional diagnoses, such as smoking: “Additional things like smoking and alcohol status are used a lot, but they’re usually very different for every project. We should have a more uniform way of doing it, like, we’ll take that bit off the shelf and use it, and do the bespoke bit for things that need to be bespoke” (data scientist 5). If there are multiple code lists for the same condition, some participants proposed that versions be generated to describe each particular condition: “So, it would be relevant that there were multiple lists for the same condition, if you’ve got a version and way of defining a certain condition” (data scientist 4).
4. Transparency: Several participants required transparency in sharing the entire approach used in developing the code lists, including phenotyping algorithms and the methods used. They stated that if they use a code list for each comorbidity of a condition, they will build an entirely different score over the years. Therefore, transparency in the documentation of research methods would help them to know which score is the best.

##### Sustainability

1. Accessibility: Several participants needed the availability of an access control that allows access to the codes only after publication, while at the first stage of the study, researchers spent a lot of time and effort developing them, and they feared someone else could publish work faster than them using the algorithms: “There should be an option in the concept library for lists that have been published. People can develop them, but if they’re not published, you don’t have to use them” (data scientist 3).
2. Licensing: Some participants needed to know which type of license was adopted by the developers of the concept library (eg, researchers can have one that means any researcher can take it and use it, or they can have one that means researchers can use it but not for commercial purposes).
3. User community: Several participants required users to quote a reference if publishing papers based on the results (partially or completely) derived from the concept library: “If I want to use someone else’s work, I think that’s the norm, and should be in this economy. Anything, not just code. To use this, I should reference that it's based on this or other thing completely, or a part of it” (data scientist 2). Referencing helps to determine whether there is or will be an active user community for the concept library and the codes used: “It potentially would make your publication more discoverable. If there's a whole community of users using this” (data scientist 1).

#### Participants’ Recommendations to Improve Repeatable Research

Of the 14 participants, 9 (64%) suggested that the prototype concept library should be accessible both in the United Kingdom and globally and practically available to enable researchers around the world to use an web-based secure platform, which stores codes and other logic, and to encourage researchers to contribute their codes to promote research: “Should be open for the United Kingdom” (data scientist 9). However, a participant recommended that the prototype concept library should be closed at the beginning to ensure it is working and then to become opened as researchers build trust: “You might need to restrict it, to start with, to make sure it works. Otherwise, everyone will see the problems you might have” (data scientist 12). In addition to know who is using the concept library, data scientist 8 suggested that it should have request sharing followed by open sharing.

Accessibility to research data has significant potential for scientific advancement as it promotes the replication of research results and enables the use of old data in new contexts. With respect to this, some participants suggested that funders and publishers should obligate researchers to share their research data such as code lists: “Some sort of obligation by funders to share this” (data scientist 2) and “Publishers, as well” (data scientist 8).

A participant suggested the use of preauthorization of publication by journals based on the research protocol because researchers can put their protocol first, and all the limitations are actually corrected before they run the research. This approach has many advantages for both the researcher and the publisher, as it improves the quality of the output. Another participant recommended the creation of a discussion forum in the concept library to facilitate collaboration among researchers on just about any topic (eg, they can share their ideas, submit their comments, and discover new ideas): “Make it almost a forum” (data scientist 8).

#### Participants’ Perceptions of Their Current Phenotyping System

The participants mentioned several problems associated with the current phenotyping system. For example, they have to search for codes from different databases, which use different coding systems such as read codes and ICD-10 codes, and then they have to validate the selected code lists with experts in the field such as clinicians: “I have to google all of this and search what was there within the community. I have to go to CALIBER, I have to go to Manchester, or there is a work in Edinburgh University, do some work there. Do the search. I have to go there, see the ability to work, and start. It does take a lot of time. Based on my study of Google, I have to start a record, and I have to validate it, verify with other people, clinicians or researchers. It's a long process” (data scientist 9).

Although they could find some codes on the web, they still had to locate the list manually, copy it, and enter the codes into their scripts*.* Often, they might spend a few days on it, and they might miss obscure codes or even use irrelevant codes: “Starting from scratch, I would go online to see what’s available. Go into other people’s and see their code lists” (data scientist 11). With respect to this, some participants said that they preferred to use code lists that were referenced or used by other researchers.

Some participants reported that the read code lists chosen by the researchers were different from the read code lists chosen by general practitioners. For example, they found that there were some very clear codes, but they were rarely used by general practitioners: “What we get in the read code list isn’t necessarily what the GPs are recording it under” (data scientist 12). They also stated that there is a significant difference between what one general practitioner may say in a list of codes versus another: “For example, there is no single entity code for asthma. There are different entities. If you want to find specific things within asthma, there's a list of codes for them” (data scientist 2).

#### Participants’ Use and Perceptions of Existing Concept Libraries

Not all participants had previously used some of the existing concept libraries. However, most of those who used some of them expressed negative perceptions. For example, several participants stated that the concept libraries they used were not user-friendly (ie, they were difficult to use by new users): “For CALIBER*,* it seems not so user friendly. It's not easy. You have to know first. Someone needs to train you up. For new users, it's difficult to get inside CALIBER. The concept library should be easy. Someone needs to train us. Concept library should have some documentation” (data scientist 9). Therefore, training and good user documentation are required. A further problem for some participants was the inconsistency of data among various databases, which makes reuse of data quite challenging. *“*But if there is something that gets secondary and primary care involved, and there's a registry, if the definitions that are created in Manchester, how easy will it be to apply it to, for example, in Wales or Scotland, where registry is a bit different?” (data scientist 8).

Participants who did not use any of the existing concept libraries expressed different perceptions about them. For example, some participants reported that they wanted to explore available concept libraries. Others, however, expressed doubts about the quality and validity of the data stored in these concept libraries, which could prevent them from using them: “I haven’t looked at them myself, but if you go on this clinical code site and you type in diabetes, there are 50 different code lists people have put together for diabetes” (data scientist 6). Some participants stated that the main reason for not using any of the existing concept libraries is not finding a concept library that matches their studies. The developers of concept libraries may consider building a specialized library that stores code lists of a particular condition within a specific group of patients according to researchers’ needs, such as developing a concept library that specializes in chronic conditions in children.

## Discussion

### Principal Findings

Development of a concept library that meets users’ expectations is extremely useful for repeatable research (eg, researchers would be able to use archived code lists to compare studies). This study found that, although in principle, everyone felt that a digital portal containing a concept library would be very helpful, there were many requirements needed before its development. It needs to engage a wide variety of users if it is to be used (and current concept libraries are not widely used), which means that it has to be very simple (point and click) for some, but it should have the software and usability to manipulate and design phenotyping algorithms for more advanced users. In addition, it needs to have a very high-quality search engine so that it is very easy to find information, and for it to expand, there needs to be a reason for users to upload their phenotyping algorithms, which need to be very easy and quick.

This study indicated that although most of the interviewees expressed positive impressions about the idea of building a prototype concept library, approximately half of the participants expressed an interest in contributing to it. For the prototype concept library to work, researchers must engage with it and upload their codes there so that other people can use them. If researchers did not share their codes in the prototype concept library, this would usually mean an empty library. For better adoption of the prototype concept library, it is recommended that the developers consider the various facilitators for and barriers to participants sharing their work and reusing the work of others.

The findings of the focus group discussion demonstrate that facilitators for the participants’ sharing of their research methods vary across four categories: (1) personal drivers (eg, obtaining appropriate credit, such as citations)—this confirms the results of earlier studies that suggest that researchers may be motivated to share their work if sharing leads to an increase in their citations [33-35], (2) benefits for their research team (eg, sharing information to promote research within their team) [36,37], (3) benefits for their organization (eg, collaboration among researchers working within the same organization would advance their organization’s research outcomes), and (4) benefits for the research community (eg, expanding research base) [38]. With respect to this, Cragin et al [39] have stated, *“*As a research group gets larger and more formally connected to other research groups, it begins to function more like big science.*”*

There were several barriers that could inhibit the participants from sharing their research methods, such as the expected performance of the shared methods (eg, they felt that building a general phenotyping algorithm to be used by others is very difficult) [40] and lack of personal benefits such as recognition (eg, they were worried about not being referenced by researchers who used their methods). In relation to this, Molloy et al [41]. reported that researchers can be discouraged from sharing their work by fear of not obtaining sufficient credit. Therefore, a safeguard against uncredited use is necessary [42]. In addition, participants mentioned that they were afraid that their methods would be used by other researchers as their own before publication. The results of the study conducted by Huang et al [43] indicated that although most participants are interested in sharing papers related to biodiversity data, >60% of the participants were reluctant to share primary data before publication. Moreover, findings from this study correspond with other studies regarding the need to adapt impact metrics to promote data sharing [44,45] because researchers would not be able to measure the success of their methods if metrics are not available. Unless these obstacles are resolved, the sharing of data in concept libraries is unlikely to increase significantly.

Several facilitators encouraged participants to reuse research methods developed by others. They reported that reusing code lists created by other researchers would make their task much easier, save them a lot of time, and help to demonstrate that there is a justification for using such codes. These findings are consistent with those of the previous studies. For example, Anneke and Helen reported that researchers are using open research data to *“*be aware of the state of the art and not recreate the wheel, as well as access to more data and generating fresh insights*”* [46].

The results of this study indicate that more than half of the participants were not satisfied with their current system for phenotyping for several reasons, including the lack of accessibility of other researchers’ work, such as code lists, which could affect research outcomes and the fact that reusing publicly available code lists consumes a lot of time and requires lots of work [38]; lack of confidence in web-based code lists if they are not cited by other researchers; lack of availability of a consistent approach for defining covariates such as smoking; and the selected read code lists by the researchers are different from the selected read code lists by the general practitioners. It seems that their current approach lacks confidence and is time-consuming and effort-intensive.

This study demonstrates that existing concept libraries are not widely used, and most participants who used some of the existing concept libraries expressed negative impressions about them (eg, they do not provide training or user documentation, and they are difficult to use) [36-38]. Lack of knowledge of the existence of concept libraries and how to use them is generally described as an obstacle to data sharing [47]. As existing concept libraries are not used by all researchers, obstacles that inhibit researchers from using them need to be addressed when building new concept libraries.

### Strengths and Limitations

To our knowledge, this is the first study aimed at identifying the needs of various users of a concept library. The findings of this study would have a significant impact on improving the efficiency of existing concept libraries by informing their developers about the different requirements, facilitators, barriers, and recommendations of the various users. In addition, this work will greatly inform the developers of new concept libraries to improve access to and collaboration with EHRs’ routine data, which is part of an all-UK agenda, and the findings of this study will have implications for other countries working to access and share EHRs’ routine data.

This study has some limitations that should be addressed in future studies. The first limitation is that we had a time limit on how long we could talk to the participants because each one-to-one interview was given 30 minutes. As a result, the number of questions we could ask and the amount of time we could spend on each question were limited. The second limitation is that all the participants of the interviews and focus group discussion were recruited because they used the SAIL databank, a national eHealth data linkage infrastructure in Wale, so they mostly talked about the Swansea concept library in the SAIL databank. As the discussion focused on the SAIL databank, its generalization to other concept libraries was limited.

### Conclusions

In conclusion, although it may seem beneficial for researchers to reuse methods developed by others, such as code lists, some researchers who created them prefer not to share them because they worked hard to create them and would rather publish them first to ensure their academic rights, such as being referenced [48]. The major challenge is that some researchers would like to use the work of other researchers, but they do not want to contribute their work to concept libraries. Open sharing can be more difficult in the research community as researchers compete for grants, work promotions, and publication quotations [48]. They think carefully about how, when, and where to share their work as they have spent a vast amount of time and effort to develop it [47]. A solution to these issues would be to encourage researchers to contribute data to the prototype concept library in such a way that the shared data is understandable and reusable (eg, ensuring uploading of adequate documentation) for the public good rather than for personal gains.

## Abbreviations

EHR: electronic health record
CALIBER: Clinical Disease Research Using Linked Bespoke Studies and Electronic Health Records
ICD: International Classification of Diseases
SAIL: Secured Anonymized Information Linkage
SWOT: strengths, weaknesses, opportunities, and threats
